# Occurrence of ESBL-*E. coli* in feces of dairy calves and nipples of teat buckets for calves before and after automatic cleaning in two dairy farms

**DOI:** 10.1038/s41598-026-62510-6

**Published:** 2026-08-03

**Authors:** Jette F. Kleist, Jenny Walkhoff, Judith Wedemeyer, Jessica Junker, Timo Homeier-Bachmann, Lisa Bachmann

**Affiliations:** 1 Faculty of Agriculture and Food Sciences, University of Applied Sciences Neubrandenburg, Brodaer Straße 2, 17033 Neubrandenburg, Germany; 2 Friedrich-Loeffler-Institute, Institute of Epidemiology, Südufer 10, 17493 Greifswald - Insel Riems, Germany; 3 Research Institute for Farm Animal Biology (FBN), Wilhelm-Stahl-Allee 2, 18196 Dummerstorf, Germany

**Keywords:** ESBL-*E. coli*, Calves, Antimicrobial resistance, Feeding equipment, Hygiene, Environmental sciences, Microbiology

## Abstract

Extended-spectrum β-lactamase (ESBL)-producing *Escherichia (E.) coli* are highly prevalent in dairy calves. In calf housing, the feeding equipment can function as a reservoir, thereby constituting a risk factor for the dissemination of ESBL-*E. coli*. The aim of this present study was to determine if ESBL-*E. coli* can be detected on the inner surface of teat buckets for calves and to evaluate if an automatic wash system specifically designed for teat buckets is suitable to eliminate existing ESBL-*E. coli* from the surfaces. Fecal samples from calves (aged three to 14 days) and teat swabs (before and after cleaning) on two farms were analyzed. Prior to cleaning, nipple contamination was detected on both farms on different levels. Automated cleaning significantly reduced contamination (*P* < 0.001). Whole-genome sequencing revealed close genetic similarities between fecal and nipple isolates, indicating transmission between calves and feeding equipment. Although the automatic cleaning significantly reduced bacterial contamination in teat buckets, ESBL-*E. coli* were detected in all calves (40/40), suggesting additional farm-level transmission routes.

**Supplementary Information:**

The online version contains supplementary material available at 10.1038/s41598-026-62510-6.

## Introduction

Antimicrobial resistance (AMR) is a major global health concern, affecting both human and veterinary medicine^1^. Within the One Health framework, extended-spectrum-β-lactamase (ESBL)-producing *Enterobacterales* are of particular concern due to their resistance to broad-spectrum-β-lactamases such as third- and fourth-generation cephalosporins^2,3^. Third-generation cephalosporin-resistant *Enterobacterales* are listed in the critical priority category in WHO’s Bacterial Priority Pathogens List (BPPL) due to their widespread prevalence and high level resistance, constituting the most significant burden among all multidrug-resistant (MDR) Gram-negative bacteria^4^. One of the most notable representatives of this group are ESBL-producing *Escherichia (E.) coli*. Their presence in livestock, particularly in dairy cattle, highlights the necessity to comprehend their transmission pathways and their persistence in livestock environments^5–7^.

The prevalence of ESBL-*E. coli* in dairy herds has been shown to be age-dependent with higher incidences observed in pre-weaned calves, which makes calves important reservoirs of ESBL-*E. coli*^5^^6^. Therefore, understanding the role of environmental transmission in early colonization is essential. Several studies have investigated risk factors that contribute to the occurrence of ESBL-*E. coli* in calves^5,8–11^. While factors such as feeding of waste milk containing antibiotic residues and antibiotic dry-off therapy in cows have been associated with an increased prevalence of ESBL-*E. coli* in calves^5,8,10^, environmental factors also play a crucial role. The dissemination of AMR bacteria can occur through direct contact with contaminated housing surfaces, bedding and feeding equipment, thereby facilitating horizontal transmission within the herd^5,8,12,13^, creating a reservoir for persistent bacterial circulation. Contaminated feeding equipment, such as teat buckets, may contribute to the spread of resistant strains within farms^5,8,12,13^, posing risks to both animal and public health. Therefore, investigating bacterial contamination in calf housing is essential for enhancing hygiene management practices and reducing the dissemination of AMR within the One Health framework. Studies have demonstrated that the hygiene of teat buckets is a prevalent issue on dairy farms^12,14^. Many farms fail to implement adequate cleaning protocols, which are crucial for ensuring the cleanliness of these buckets. This is often due to the significant time investment necessary for comprehensive cleaning procedures. Given the critical importance of good teat bucket hygiene, not only in terms of ESBL-*E. coli* colonization but also for the general health of calves, practical solutions are required. One approach is the utilization of an automatic wash system designed for the cleansing of teat buckets^15^.

The present study aimed to determine if ESBL-*E. coli* can be detected on the inner surface of teat buckets for calves and to evaluate if an automatic wash system specifically designed for teat buckets is suitable to eliminate existing ESBL-*E. coli* from the surfaces and, is therefore suitable to minimize the prevalence of ESBL-*E. coli* in dairy calves.

## Material and Methods

### GRETE system for automatic cleaning of teat buckets

GRETE is a machine for automatic cleaning of teat buckets from the manufacturer “GRETE GmbH” (Illertissen, Germany). Up to 10 teat buckets can be cleaned in the machine at the same time. Buckets are placed upside down and artificial teats are placed between a plastic roller and a metal plate so that the teats can be wiped out by rotating the rollers. This allows milk residue and water to be rinsed through the entire teat. Teats are cleaned and drained 12 times per minute. With a cleaning time of 15–20 min, this takes place up to 200 times per wash cycle. First, the buckets are pre-rinsed with cold water before hot water and acidic and alkaline cleaners are added. The manufacturers of the GRETE system recommend using acidic and alkaline cleaning agents that are typically intended for milking parlors. Products of Finktec GmbH were used and applied in accordance with the manufacturer’s instructions. For the study, the washing programme with the alkaline cleaning agents was chosen. Differently positioned nozzles ensure cleaning inside and outside the bucket. Buckets are tilted several times during wash cycles to rinse out residues. At the end, buckets are rinsed several times with cold water^15–17^.

### Farms and calf management

This study included two conventional German dairy farms that use the GRETE system for automatic teat bucket cleaning. Data on calf housing, feeding and teat bucket hygiene were collected. The farms were selected because they both use the GRETE system, which not many farms have adopted yet.

Farm 1 milks 2,800 Holstein cows, while farm 2 milks 600. Calves are kept in individual hutches for either 10 days (farm 1) or 21 days (farm 2), and fed pasteurized whole milk via teat buckets three times a day (portioned, three times/day, farm 1) or twice a day (*ad libitum*, twice/day, farm 2). Despite contrary recommendation, the whole milk fed to the calves on both farms is waste milk, which may contain antibiotic residues.

On farm 1, most teat buckets are cleaned once daily using the GRETE system. For the other two feedings, they are hand-washed with warm water and an alkaline cleaner. Buckets are reused immediately. Approximately 200 buckets are available for up to 180 calves and buckets are only replaced when severely damaged.

On farm 2, all teat buckets are cleaned daily with the GRETE system. Unlike on farm 1, the buckets are hung up to dry completely until at least the next day before reuse. Approximately 100 buckets are available for up to 64 calves, enabling overnight drying. Every five weeks, the buckets are unscrewed and cleaned manually with warm water, an alkaline cleaner and a brush. They are then fitted with new teats.

### Sampling, bacteriological examination

60 swab samples (swabs containing 1 mL Amies transport medium, SIGMA TRANSWAB, MWE, United Kingdom) were taken per farm for the determination of ESBL-*E. coli*. On each farm, 20 fecal swabs were taken from calves, and further 20 swab samples were taken from the inside of the buckets’ teats before and after cleaning with the GRETE system. Teat buckets assigned to the calves were sampled. Holstein calves were between three and 14 days old and the sex ratio was balanced. Informed consent and permission for sample collection were obtained from the respective farm operators prior to sampling of client-owned dairy calves.

After sampling, samples were stored under refrigeration until transport to the laboratory. Samples were transported without refrigeration and the transport time was approximately one hour. Teat bucket samples were cultured in 5 mL lysogeny broth (LB) medium (Carl Roth GmbH, Karlsruhe, Germany) supplemented with 2 µg/mL cefotaxime (VWR International, Darmstadt, Germany) and incubated overnight at 37 °C before being plated on CHROMagar™ Orientation agar plates (MAST Group, Reinfeld, Germany) supplemented with 2 µg/mL cefotaxime (VWR International, Darmstadt, Germany) and incubated overnight at 37 °C. Fecal samples were directly cultured on the agar plates. Potential ESBL-*E. coli* colonies (pink-violet colored) were sub-cultivated until pure cultures were obtained.

### Antimicrobial susceptibility testing (AST)

AST was performed with VITEK2 (bioMérieux, Nürtingen, Germany) using software version 9.02 and AST-N389 card, according to manufacturer’s instructions. First-, third-, fourth-, and fifth-generation cephalosporins cefalexine, cefotaxime, ceftazidime, ceftolozane and cefepime were tested individually or in combination with tazobactam. If bacterial growth is inhibited in combination with tazobactam, VITEK2 classifies the isolates as possible ESBL producers. If no growth inhibition occurs upon the addition of β-lactamase inhibitors, VITEK2 classifies them as AmpC producers. AST results of VITEK2 system were interpreted based on the European Committee on Antimicrobial Susceptibility Testing (EUCAST) breakpoint tables for interpretation of MICs and zone diameters (Version 9.02, 2021. https://www.eucast.org/).

### Sequence analysis

DNA extraction was performed with MasterPure™ DNA Purification Kit for Blood, Version II (Lucigen, Middleton, USA). DNA samples of all bacterial strains were then shipped to SeqCenter (Pittsburgh, PA, USA) and subjected to whole-genome-sequencing (WGS) as described elsewhere^18^.

### Clonal expansion

For 57 isolates (ST10, ST88, ST362, ST536 and ST1434), clonal expansion analysis was performed to construct their core single nucleotide polymorphisms (SNP) phylogeny. WGS reads were aligned to reference genomes (3189/ST10,3191/ST88, 3192/ST362, 3193/ST536 and 3190/ST1434) using Snippy v.4.6.0 (https://github.com/tseemann/snippy). The alignment was then processed using Gubbins^19^ v.2.4.1 and snp-sites^20^ v.2.5.1. Maximum-likelihood phylogenetic trees were inferred from these alignments using FastTree^21^ v.2.1.11 (http://www.microbesonline.org/fasttree/). Midpoint-rooting of the trees was carried out in iTOL^22^ v.7.0. To convert the FASTA alignment to a SNP distance matrix, Snp-dists v.0.8.2 (https://github.com/tseemann/snp-dists) was used. A cutoff of 17 SNPs was applied to all STs to identify transmission between calves and teat buckets^23^. The resulting matrix was visualized as a heat map diagram.

### Biofilm formation

The biofilm-forming capacity of *E. coli* isolates was assessed using a crystal violet staining assay adapted from O´Toole 2011^24^ and Cirrincione et al.^25^ with slight modification. All isolates were tested in triplicate. A total of 100 µL of a suspension in the logarithmic growth phase was transferred into each well of a 96-well microplate and incubated at 37 °C for 48 h. After incubation, the optical density (OD) was measured at 540 nm using a microplate reader (Tecan Trading AG, Switzerland), and the suspension was carefully removed. Subsequently, 0.1% crystal violet was added and incubated for 30 min at room temperature, followed by three washing steps with 1× PBS. The deposits at the bottom of the wells were then air-dried and subsequently dissolved in 30% acetic acid and incubated for 20 min. The resulting solution was transferred to a new microtiter plate, and the absorbance was measured at 595 nm. The capacity was determined based on the ratio of OD_595mm_ to OD_540mm_. Based on the results, the isolates were classified into different categories as described by Stepanović et al.^26^.

The assay was performed alongside the reference strain *E. coli* DH5α and a negative control containing LB-medium only.

### Statistical methods

A Chi-square test was performed in Microsoft Excel (Version 2021) to ascertain if cleaning the teat buckets with the GRETE system is able to significantly reduce ESBL-*E. coli* from the inner surface of the nipples. Due to the small sample size, the calculation was not performed at farm level but rather as an aggregate result for both farms.

## Results

### Isolates

In both farms, all fecal samples of the calves (*n* = 20 per farm) were tested positive for ESBL-*E. coli*. However, the load of ESBL-*E. coli* in the nipples of teat buckets was different between herds. Prior to cleaning, all 20 swabs (100%) collected from the inside of the nipples on farm 1 were tested positive for ESBL-*E. coli*, while only one sample (5%) from farm 2 exhibited a positive result. After cleaning with GRETE, six nipples (30%) on farm 1 remained positive, whereas in farm 2 all nipples were tested negative for ESBL-*E. coli*. A total, of 67 ESBL-*E. coli* isolates were obtained.

**Table 1:** Number of ESBL-*E. coli* isolates of samples from calves’ feces, nipples before and after automatic cleaning.

Origin of samples (*n* = 20)	Number of positive samples		
	Farm1	Farm 2	Total
Feces of calves	20	20	40
Nipples before cleaning	20	1	21
Nipples after cleaning	6	0	6*

*Statistically significant different from number of ESBL-*E. coli* positive nipples before cleaning (*P* < 0.001).

### Antimicrobial susceptibility testing (AST)

The results of the AST are shown in Supplementary Table 1. Among the 67 isolates examined, 66 exhibited an ESBL-phenotype, indicative of resistance to amoxicillin, amoxicillin/clavulanic acid, ampicillin, cefalexin, cefotaxime, ceftazidime and cefepime and less resistant behavior against combinations with β-lactamase inhibitor tazobactam. One isolate (3255, feces, farm 1) exhibited an AmpC-phenotype due to a sensitivity for cefotaxime and demonstrated resistance to the last-resort antibiotic colistin. A total of 22 isolates (32.8%) were identified as multidrug-resistant (MDR), i.e. exhibited resistances towards at least three antimicrobial classes^27^. On farm 1, 11 MDR-isolates were identified, with seven being isolated from fecal samples and two each from nipples before and after cleaning. In each instance, the fecal and nipple samples were obtained from different calves. All 11 MDR-isolates from farm 2 originated from fecal samples, none were found in nipples.

### Whole-genome sequencing

All isolates underwent WGS. Details of WGS are shown in Supplementary Table 2. MLS typing identified 11 sequence types (STs) The presence of three STs (ST10, ST88 and ST362) was observed on both farms, while ST16, ST117, ST536, ST1434, ST1571 and ST5295 were exclusively detected on farm (1) ST58 and ST2325 were exclusively found in farm (2) Following the cleaning process, the following STs remained present in the nipples: ST10, ST362, ST536, ST1434 and ST1571.

WGS confirmed all isolates as ESBL-*E. coli* since ESBL genes were present in all 67 isolates. The majority of the isolates harbored *bla*_*CTX−M−1*_, (57/67 = 85%), while *bla*_*CTX−M−15*_ (8/67 = 12%), *bla*_*CTX−M−65*_ (2/67 = 3%), *bla*_*TEM−1 A*_ (11/67 = 16%), *bla*_*TEM−1B*_ (6/67 = 9%) and *bla*_*OXA−1*_ (5/67 = 7%) were also identified. The most frequently represented gene was *mdf* (67/67), which encodes a transport protein providing low level resistance to a wide range of antibiotics and toxic substances^8^. Nineteen isolates (28%) carried the *qnrS1* gene associated with quinolone resistance. Additionally, several aminoglycoside, phenicol, trimethoprim, lincosamide, macrolide, tetracycline and sulfonamide resistance genes were identified (see Supplementary Table 2).

### Clonal expansion

**Fig. 1:**
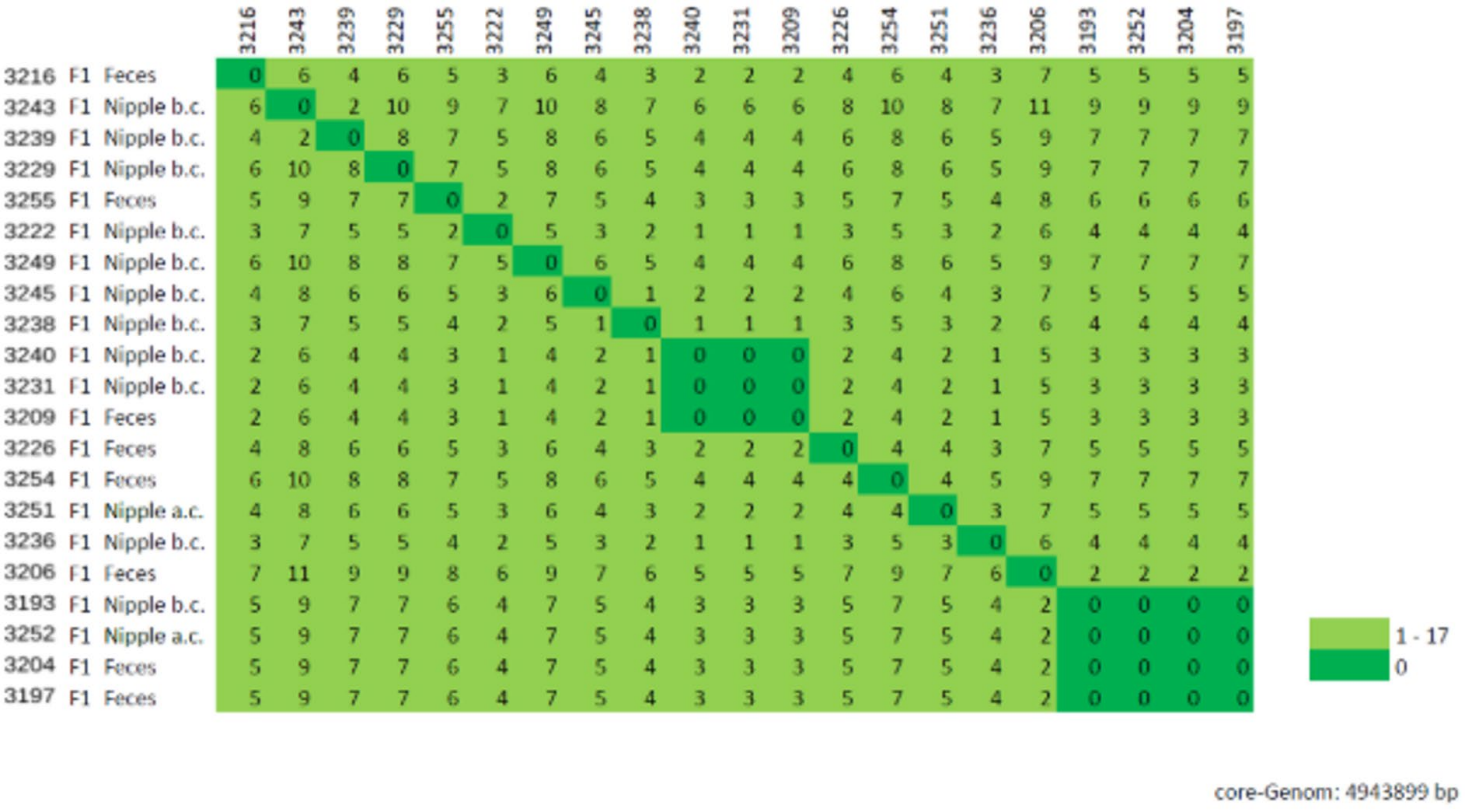
Core SNP phylogeny of ST536 isolates. F1: farm 1. b.c.: before cleaning. a.c.: after cleaning.

Phylogenetic analysis of ST10, ST88, ST362 (farms 1 and 2) ST536 and ST1434 (farm 1) based on WGS data revealed close genetic similarities between some of the isolates of the respective ST. Heat map diagrams of all STs analyzed are shown in the supplement. The core genome size of the ST536 isolates under study was determined to be 4,943,899 bp. A close genetic similarity was revealed for all 21 isolates, with a maximum variation in base pairs of 11 SNPs within the core genome (Fig. 1). The diagram includes fecal isolates as well as isolates collected from teat before and after the cleaning process. Analysis of the other STs yielded analogous results, as illustrated in the Supplementary Figs. 1–4.

### Biofilm formation

**Fig. 2:**
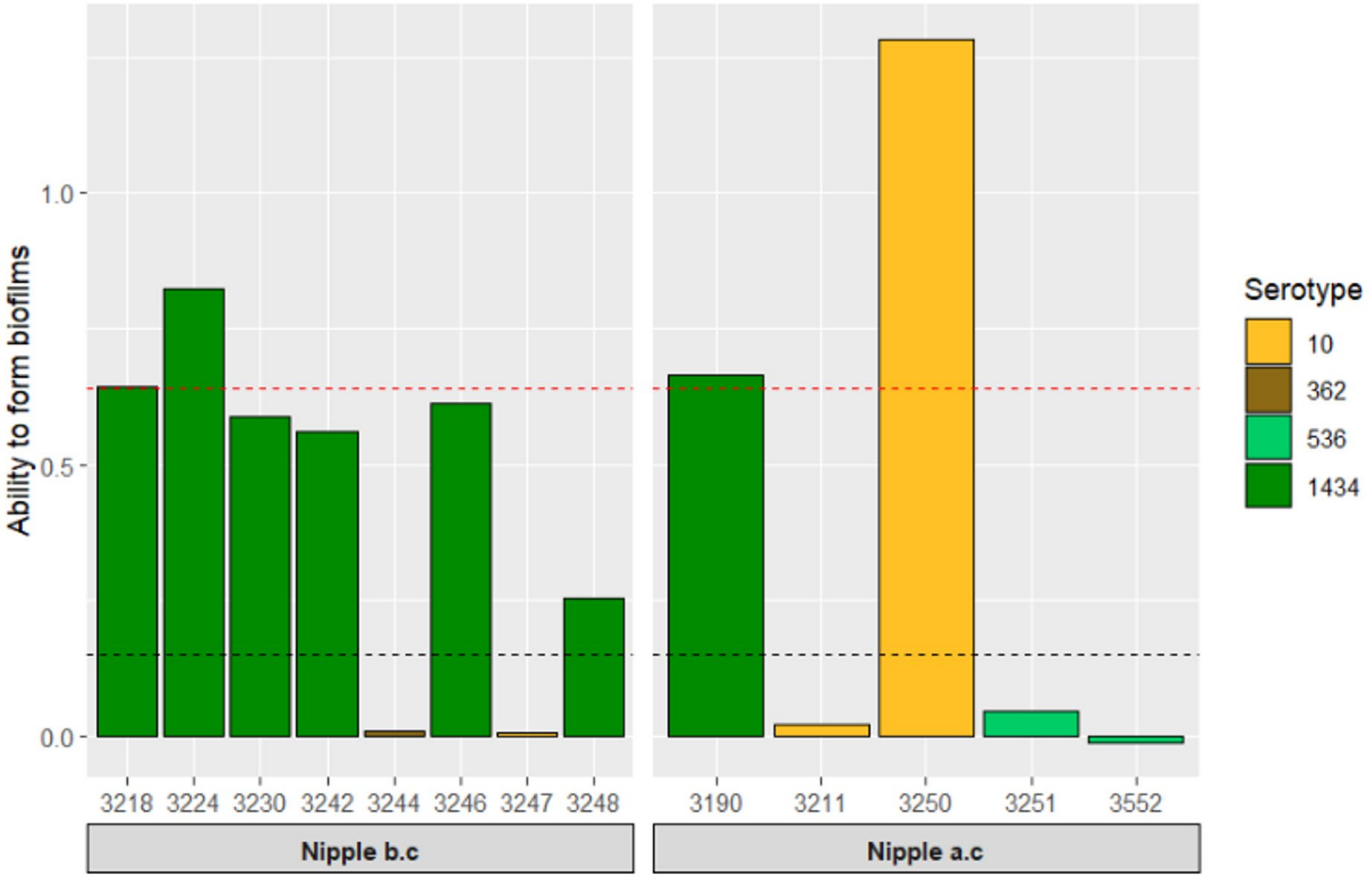
Testing 13 ESBL-positive *E. coli* isolates for their ability to form biofilms using a crystal violet assay. The ability was calculated by the ration of OD595mm und OD540mm. Black dotted line: Cut-Off-Value by 0.149. Red dotted linie: threshold for strong biofilm former. b.c.: before cleaning. a.c.: after cleaning.

To test the ability to form biofilms, 13 isolates were selected from nipples before and after cleaning. The isolates were selected from strains identified, based on WGS results, as those likely to have been transmitted between calves and feeding buckets and represent the clonal clusters. In both groups, isolates exhibited considerable variability in biofilm formation, ranging from no detectable biofilm production to strong biofilm-forming capacity (see Fig. 2).

## Discussion

It is a globally acknowledged issue that livestock production plays a significant role in the spread of AMR^28,29^. Within the context of dairy farming, suckling calves exhibit the highest prevalence of ESBL-*E. coli*, often exceeding 50%, whereas older heifers and adult cows generally show significantly lower prevalence rates^5,6,30^. Widespread practices in calf feeding, such as the feeding of waste milk^5,31^ or insufficient cleaning of teat buckets, which are recognized as reservoirs of bacteria^5,12^, are amongst the numerous risk factors to which calves are exposed in relation to ESBL*-E. coli*.

All fecal samples from calves in the present study were tested positive for ESBL-*E. coli*, indicating a consistent colonization of calves on both farms during the first two weeks of life. This result aligns with the findings of other studies that have reported high prevalence of ESBL-*E. coli* in suckling calves^5,6,10^. Given the recognized propensity of feeding equipment to function as a reservoir for ESBL-*E. coli*^5^^12^, the following observations were anticipated. Before cleaning with the GRETE system, all 20 teat buckets on farm 1 were tested positive for ESBL-*E. coli*, while this condition was only observed in one teat bucket (1/20) on farm 2, even though all teat buckets belonged to calves colonized with ESBL-*E. coli*. Following the cleaning procedure, the nipples from farm 2 demonstrated no contamination with ESBL-*E. coli*, whereas the nipples from farm 1 demonstrated residual ESBL-*E. coli* presence, as evidenced by the colonization of six teat buckets (see Table 1). However, the GRETE system demonstrated a statistically significant reduction in the prevalence of ESBL-*E. coli*-positive nipples (*P* < 0.001). To find out whether biofilms could be a reason for the remaining isolates in the cleansed nipples, the capacity of biofilm formation was tested. The testing of isolates originiating from teats before and after cleaning revealed that certain isolates exhibited a high capacity to form biofilms. Among the 13 isolates that were tested, seven were found to be strong biofilm builders. Two of the seven isolates were obtained from teats after cleaning with the GRETE system.

For three isolates representing the clonal clusters from teats after cleaning on farm 1, no ability to form biofilms could be detected, which leads to the suggestion, that these isolates are adapted to persist on the surface otherwise. The sole isolate detected within a teat on farm 2 before the cleaning procedure exhibited no capacity to form biofilms. The observed variations in the daily handling of the teat buckets across the two farms may be indicative of the overall minimal colonization of the teat buckets on farm 2. Following the cleaning process, the buckets are hung up to thoroughly dry on farm 2, whereas this is not the case on farm 1. As indicated by the findings of numerous studies, the survival rate of *E. coli* on dry surfaces is notably diminished due to its sensitivity to desiccation^32,33^. One possible explanation for these observations is that complete drying of the teat buckets on farm 2 limits the survival of *E. coli* remaining on the nipples after cleaning. In contrast, the persistently moist conditions of the teat buckets in on farm 1 may promote bacterial survival, thereby allowing *E. coli* not eliminated during the cleaning process to persist between uses. Additionally, it should be noted that not all teat buckets at farm 1 are cleaned with the GRETE system on a daily basis. Due to the limited capacity of the GRETE-system, a number of the buckets are instead cleaned manually by hand, a method that has been demonstrated to be a risk factor for the dissemination of germs on the buckets due to the carryover of germs via the washing water^5^. On farm 2, the teat buckets undergo a dual cleaning process. The GRETE-system is utilized for daily cleaning, while manual cleaning is conducted at a five week-interval, involving the unscrewing of the teats from the buckets. According to Heinemann et al., the unscrewing and separate cleaning increases the effectiveness of the cleaning process in terms of reducing bacterial contamination^12^. The findings indicate that, although the GRETE system is capable of significantly reducing bacterial contamination in the teat buckets, the presence of ESBL-*E. coli* in all calves tested points to additional sources and transmission routes on the farms that contribute to the prevalence of ESBL-*E. coli* in calves. In this regard, on-farm practices known to be risk factors for the occurrence of ESBL-*E. coli* were identified. On both farms, calves are fed waste milk, which may contain antibiotic residue. Feeding waste milk is considered one of the greatest risks for ESBL-*E. coli* in calves^5^. Since cows on both farms undergo antibiotic dry cow therapy, this risk is further increased^34.^ In the present study, waste milk was not subjected to testing for the presence of ESBL-*E. coli.* Consequently, any potential influence of waste milk on the prevalence of ESBL*-E. coli* in both calves and teat buckets tested in this study can only be hypothesized. Furthermore, on both farms, calves are fed colostrum, which is collected in a milking pot. Bachmann et al. identified these pots as a possible source of ESBL-*E. coli* in calves^8^. These observations highlight potential sources and transmission routes of ESBL-*E. coli* in both farms that also need improvement, in order to ensure the efficacy of the GRETE system. The presence of the STs ST10, ST88 and ST362 was identified in both farms. In the context of dairy farming, those STs are frequently referenced^6,8^. Clonal expansion analysis revealed that clones of the isolates belonging to said STs from the fecal swabs were also present in the teat buckets. A similar observation was made with isolates belonging to ST536 and ST1434, which were detected in farm 1 in feces and teat buckets. All isolates belonging to these STs meet the criteria for clone definition by Ludden et al. ^23^, thereby indicating a direct transmission pathway between calves and their feeding equipment. The presence of isolates belonging to specific STs, including ST10, ST58, ST88, ST117 and ST362, is of concern due to their documented global prevalence as extraintestinal pathogenic *E. coli* (ExPEC) lineages associated with both human and veterinary diseases^35–37^. It is widely acknowledged that bacteria are transmitted among humans and animals^7,38,39^. Consequently, the presence of potentially pathogenic strains in livestock populations poses a potential risk to human health. The WGS findings were consistent with those expected in the context of calf housing, with the identification of CTX-M-genes *bla*_CTX−M−1_, *bla*_CTX−M−15_ and *bla*_CTX−M−65_ in the majority of the isolates within the two farms^5,6,8,10^. Of particular concern is the finding that 22 isolates (32.8%) are classified as MDR with one isolate (3255) being resistant against the last-resort antibiotic colistin. In contrast to the prevailing definition of ESBL, which necessitates susceptibility to drug combinations containing β-lactamase inhibitors such as clavulanic acid^40^, all isolates demonstrated resistant behavior to amoxicillin/clavulanic acid. Notwithstanding this phenotypic resistance, WGS confirmed all isolates as ESBL-*E. coli*. This phenomenon of divergence between phenotype and genotype has been observed before^41^.

## Conclusion

The present study confirmed the role of teat buckets as a risk factor in the spread of ESBL-*E. coli* on dairy farms. The surface of the nipples was identified as a reservoir for these bacteria, and clones of ESBL-*E. coli*-STs were found in the nipples and in the feces of the calves suggesting transmission between nipples and the calves. The study also demonstrated that the utilization of an automatic washing system, such as the GRETE-system, to clean teat buckets used for calf feeding, can significantly reduce the colonization of said buckets with ESBL-*E. coli*. The observed variations in outcomes on farm level, in conjunction with the heterogeneity in on-farm practices, indicate that the daily handling of the teat buckets exerts a substantial influence on contamination levels. This indicates that achieving complete elimination of ESBL-*E. coli* in teat buckets is not achievable through the implementation of the GRETE-system alone. Based on our findings, which suggest reduced bacterial persistence in completely dried teat buckets, additional adjustments to the daily handling of the teat buckets, such as complete drying before further feedings, are needed to eliminate ESBL-*E. coli* completely. Although GRETE reduced the ESBL-*E. coli* load in the nipples of teat buckets, more intervention measures to reduce are necessary to decrease the prevalence of ESBL-*E. coli* in dairy calves.

## Electronic Supplementary Material

Below is the link to the electronic supplementary material.

Supplementary Material 1

Supplementary Material 2

Supplementary Material 3

Supplementary Material 4

Supplementary Material 5

Supplementary Material 6

## Data Availability

The datasets generated and analysed within this study are available in the European Nucleotide Archive repository with the primary accession code PRJEB111890.
