# Supplementary material for: Occurrence of ESBL-E. coli in feces of dairy calves and nipples of teat buckets for calves before and after automatic cleaning in two dairy farms
Source: Sci Rep. 2026 Aug 3;16:23886. doi: 10.1038/s41598-026-62510-6 (PMC13434752; doi:10.1038/s41598-026-62510-6)
Supplement: Supplementary file 1 [file 41598_2026_62510_MOESM2_ESM.docx]

| Designation | Farm | Calf | Origin of Sample | ESBL | MDR | Amoxicillin | Ampicillin | Amoxicillin/clavulanic acid | Piperacillin/tazobactam | Cefalexine | Cefotaxime | Ceftazidime | Ceftolozane/tazobactam | Cefepim | Aztreonam | Imipenem | Meropenem | Amikacin | Gentamicin | Tobramycin | Ciprofloxacin | Tigecyclin | Fosfomycine | Colistin | Trimethoprim/sulfamethoxazole |
| --- | --- | --- | --- | --- | --- | --- | --- | --- | --- | --- | --- | --- | --- | --- | --- | --- | --- | --- | --- | --- | --- | --- | --- | --- | --- |
| 3198 | 1 | 20018 | Fecal | + |  | R | R | R | I | R | R | R | S | R | I | S | S | S | S | S | S | S | S | S | S |
| 3224 | 1 | 20018 | Nipple b. c. | + |  | R | R | R | I | R | R | R | S | R | I | S | S | S | S | S | S | S | S | S | S |
| 3252 | 1 | 20018 | Nipple a. c. | + |  | R | R | R | I | R | R | R | S | R | I | S | S | S | S | S | S | S | S | S | S |
| 3213 | 1 | 22007 | Fecal | + |  | R | R | R | I | R | R | R | S | R | I | S | S | S | S | S | S | S | S | S | S |
| 3193 | 1 | 22007 | Nipple b. c. | + |  | R | R | R | I | R | R | R | S | R | I | S | S | S | S | S | S | S | S | S | S |
| 3255 | 1 | 22011 | Fecal |  | + | R | R | R | R | R | S | R | R | I | R | S | S | S | S | S | S | S | S | R | S |
| 3245 | 1 | 22011 | Nipple b. c. | + |  | R | R | R | I | R | R | R | S | R | I | S | S | S | S | S | S | S | S | S | S |
| 3254 | 1 | 22013 | Fecal | + |  | R | R | R | I | R | R | R | S | R | I | S | S | S | S | S | S | S | S | S | S |
| 3231 | 1 | 22013 | Nipple b. c. | + |  | R | R | R | I | R | R | R | S | R | I | S | S | S | S | S | S | S | S | S | S |
| 3253 | 1 | 22013 | Nipple a. c. | + | + | R | R | R | I | R | R | R | S | R | S | S | S | S | S | S | S | S | S | S | R |
| 3214 | 1 | 22015 | Fecal | + | + | R | R | R | I | R | R | R | S | R | I | S | S | S | S | S | S | S | S | S | R |
| 3236 | 1 | 22015 | Nipple b. c. | + |  | R | R | R | I | R | R | R | S | R | I | S | S | S | S | S | S | S | S | S | S |
| 3200 | 1 | 22017 | Fecal | + | + | R | R | R | I | R | R | R | S | R | S | S | S | S | S | S | S | S | S | S | R |
| 3248 | 1 | 22017 | Nipple b. c. | + |  | R | R | R | I | R | R | R | S | R | I | S | S | S | S | S | S | S | S | S | S |
| 3205 | 1 | 22018 | Fecal | + | + | R | R | R | I | R | R | R | S | R | S | S | S | S | S | S | S | S | S | S | R |
| 3247 | 1 | 22018 | Nipple b. c. | + |  | R | R | R | I | R | R | R | S | R | I | S | S | S | S | S | S | S | S | S | S |
| 3190 | 1 | 22018 | Nipple a. c. | + |  | R | R | R | I | R | R | R | S | R | I | S | S | S | S | S | S | S | S | S | S |
| 3204 | 1 | 22022 | Fecal | + |  | R | R | R | I | R | R | R | S | R | I | S | S | S | S | S | S | S | S | S | S |
| 3218 | 1 | 22022 | Nipple b. c. | + |  | R | R | R | I | R | R | R | S | R | I | S | S | S | S | S | S | S | S | S | S |
| 3202 | 1 | 22025 | Fecal | + | + | R | R | R | I | R | R | R | S | R | R | S | S | S | R | R | R | S | S | S | R |
| 3222 | 1 | 22025 | Nipple b. c. | + |  | R | R | R | I | R | R | R | S | R | I | S | S | S | S | S | S | S | S | S | S |
| 3211 | 1 | 22025 | Nipple a. c. | + |  | R | R | R | I | R | R | R | S | R | I | S | S | S | S | S | S | S | S | S | S |
| 3201 | 1 | 22029 | Fecal | + |  | R | R | R | I | R | R | R | S | R | I | S | S | S | S | S | S | S | S | S | S |
| 3249 | 1 | 22029 | Nipple b. c. | + |  | R | R | R | I | R | R | R | S | R | I | S | S | S | S | S | S | S | S | S | S |
| 3197 | 1 | 24009 | Fecal | + |  | R | R | R | I | R | R | R | S | R | I | S | S | S | S | S | S | S | S | S | S |
| 3241 | 1 | 24009 | Nipple b. c. | + | + | R | R | R | I | R | R | R | S | R | I | S | S | S | S | S | S | S | S | S | R |
| 3226 | 1 | 24192 | Fecal | + |  | R | R | R | I | R | R | R | S | R | I | S | S | S | S | S | S | S | S | S | S |
| 3229 | 1 | 24192 | Nipple b. c. | + |  | R | R | R | I | R | R | R | S | R | I | S | S | S | S | S | S | S | S | S | S |
| 3223 | 1 | 24194 | Fecal | + |  | R | R | R | I | R | R | R | S | R | I | S | S | S | S | S | S | S | S | S | S |
| 3243 | 1 | 24194 | Nipple b. c. | + |  | R | R | R | I | R | R | R | S | R | I | S | S | S | S | S | S | S | S | S | S |
| 3250 | 1 | 24194 | Nipple a. c. | + | + | R | R | R | I | R | R | R | S | R | I | S | S | S | R | R | R | S | S | S | R |
| 3234 | 1 | 24195 | Fecal | + | + | R | R | R | I | R | R | R | S | R | R | S | S | S | S | S | S | S | S | S | S |
| 3242 | 1 | 24195 | Nipple b. c. | + |  | R | R | R | I | R | R | R | S | R | I | S | S | S | S | S | S | S | S | S | S |
| 3232 | 1 | 24198 | Fecal | + | + | R | R | R | I | R | R | R | S | R | S | S | S | S | S | S | S | S | S | S | R |
| 3246 | 1 | 24198 | Nipple b. c. | + |  | R | R | R | I | R | R | R | S | R | I | S | S | S | S | S | S | S | S | S | S |
| 3217 | 1 | 24205 | Fecal | + |  | R | R | R | I | R | R | R | S | R | I | S | S | S | S | S | S | S | S | S | S |
| 3230 | 1 | 24205 | Nipple b. c. | + |  | R | R | R | I | R | R | R | S | R | I | S | S | S | S | S | S | S | S | S | S |
| 3233 | 1 | 24206 | Fecal | + |  | R | R | R | I | R | R | R | S | R | I | S | S | S | S | S | S | S | S | S | S |
| 3238 | 1 | 24206 | Nipple b. c. | + |  | R | R | R | I | R | R | R | S | R | I | S | S | S | S | S | S | S | S | S | S |
| 3251 | 1 | 24206 | Nipple a. c. | + |  | R | R | R | I | R | R | R | S | R | I | S | S | S | S | S | S | S | S | S | S |
| 3206 | 1 | 24209 | Fecal | + |  | R | R | R | I | R | R | R | S | R | I | S | S | S | S | S | S | S | S | S | S |
| 3239 | 1 | 24209 | Nipple b. c. | + |  | R | R | R | I | R | R | R | S | R | I | S | S | S | S | S | S | S | S | S | S |
| 3209 | 1 | 24216 | Fecal | + |  | R | R | R | I | R | R | R | S | R | I | S | S | S | S | S | S | S | S | S | S |
| 3240 | 1 | 24216 | Nipple b. c. | + |  | R | R | R | I | R | R | R | S | R | I | S | S | S | S | S | S | S | S | S | S |
| 3216 | 1 | 24219 | Fecal | + |  | R | R | R | I | R | R | R | S | R | I | S | S | S | S | S | S | S | S | S | S |
| 3227 | 1 | 24219 | Nipple b. c. | + | + | R | R | R | I | R | R | R | S | R | S | S | S | S | S | S | S | S | S | S | R |
| 3219 | 2 | 14331 | Fecal | + | + | R | R | R | I | R | R | R | S | R | I | S | S | S | S | S | I | S | S | S | R |
| 3237 | 2 | 14332 | Fecal | + | + | R | R | R | R | R | R | R | S | R | I | S | S | S | S | S | I | S | S | S | R |
| 3235 | 2 | 14334 | Fecal | + |  | R | R | R | I | R | R | R | S | R | I | S | S | S | S | S | S | S | S | S | S |
| 3244 | 2 | 14334 | Nipple b. c. | + |  | R | R | R | I | R | R | R | S | R | I | S | S | S | S | S | S | S | S | S | S |
| 3225 | 2 | 14335 | Fecal | + | + | R | R | R | I | R | R | R | S | R | R | S | S | S | S | S | S | S | S | S | S |
| 3212 | 2 | 14336 | Fecal | + | + | R | R | R | R | R | R | R | S | R | I | S | S | S | S | S | S | S | S | S | R |
| 3194 | 2 | 14337 | Fecal | + | + | R | R | R | I | R | R | R | S | R | I | S | S | S | S | S | S | S | S | S | R |
| 3208 | 2 | 14338 | Fecal | + | + | R | R | R | I | R | R | R | S | R | R | S | S | S | S | S | S | S | S | S | S |
| 3196 | 2 | 14339 | Fecal | + |  | R | R | R | I | R | R | R | S | R | I | S | S | S | S | S | S | S | S | S | S |
| 3210 | 2 | 14340 | Fecal | + |  | R | R | R | I | R | R | R | S | R | I | S | S | S | S | S | S | S | S | S | S |
| 3199 | 2 | 14341 | Fecal | + | + | R | R | R | I | R | R | R | S | R | R | S | S | S | S | S | R | S | S | S | S |
| 3189 | 2 | 60782 | Fecal | + |  | R | R | R | I | R | R | R | S | R | I | S | S | S | S | S | I | S | S | S | R |
| 3228 | 2 | 60787 | Fecal | + | + | R | R | R | R | R | R | R | S | R | I | S | S | S | S | S | S | S | S | S | R |
| 3191 | 2 | 60789 | Fecal | + | + | R | R | R | I | R | R | R | S | R | R | S | S | S | S | S | R | S | S | S | S |
| 3207 | 2 | 60794 | Fecal | + |  | R | R | R | I | R | R | R | S | R | I | S | S | S | S | S | S | S | S | S | S |
| 3203 | 2 | 60795 | Fecal | + | + | R | R | R | I | R | R | R | S | R | R | S | S | S | S | S | S | S | S | S | S |
| 3195 | 2 | 60796 | Fecal | + |  | R | R | R | I | R | R | R | S | R | I | S | S | S | S | S | S | S | S | S | S |
| 3220 | 2 | 60797 | Fecal | + | + | R | R | R | I | R | R | R | S | R | I | S | S | S | S | S | S | S | S | S | R |
| 3215 | 2 | 60802 | Fecal | + |  | R | R | R | I | R | R | R | S | R | I | S | S | S | S | S | S | S | S | S | S |
| 3221 | 2 | 60803 | Fecal | + |  | R | R | R | I | R | R | R | S | R | I | S | S | S | S | S | S | S | S | S | S |
| 3192 | 2 | 60804 | Fecal | + |  | R | R | R | I | R | R | R | S | R | I | S | S | S | S | S | S | S | S | S | S |
